# Female Body Mass Index and its Relationship With Triple Negative Breast Cancer and Ethnicity

**DOI:** 10.4021/wjon709w

**Published:** 2013-09-27

**Authors:** Khurram Bilal Tariq, Fauzia Rana

**Affiliations:** a Department of Internal Medicine, University of Florida, College of Medicine, USA; b Department Chair, Division of Hematology and Oncology, University of Florida, College of Medicine, Jacksonville, Fl, USA

**Keywords:** BMI, Breast cancer, Triple negative breast cancer, Overweight, Obesity

## Abstract

**Background:**

Breast cancer in women is a relatively common malignancy in the western hemisphere and is perhaps one of the leading causes of mortality among females. We conducted a retrospect cohort study to investigate the association of body mass index (BMI) with triple negative breast cancer and ethnicity.

**Methods:**

Tumor Registry Database at the University of Florida, College of Medicine in Jacksonville was utilized for our cohort study. A total of 84 women with triple negative breast cancer, between 2004 and 2008 met our criteria and were selected for this study. For comparison, another 83 women with at least one hormone receptors positive breast cancer were randomly selected in the same time period. Chi-square testing was used to evaluate categorical variables, while the t-test analysis was used to analyze for the continuous variables.

**Results:**

Our data demonstrated that 27.4% of the triple negative group had BMI < 25 compared to the 14.5% of non-triple negative breast cancer, 73.6% of the triple negative group had BMI ≥ 25 compared to 86.5% in the non-triple negative group with a P-value of 0.245. In terms of ethnicity, triple negative breast cancer was found in 56% of African-American and 44% of Caucasian females. Non-triple negative breast cancer was found in 48.2% of African-Americans and 51.8% of Caucasians females with a P-value of 0.354.

**Conclusions:**

We were not able to show any statistically significant association of body mass index triple with triple negative breast cancer or ethnicity. While our findings are not in agreement with the research published earlier, we do submit that our retrospective cohort study has shortcomings, including the small sample size pooled from a single center, which greatly limits our ability to deduce any definitive conclusions. In light of these shortcomings, we recommend a future multicenter study with a larger sample size.

## Introduction

Body mass index is a modifiable risk factor that has been associated with several co-morbidities, including type 2 Diabetes, risk of cardiovascular diseases and various types of cancers, to name a few [1, 2]. This is an alarming development especially in a country such as ours, where two-third of the US population in either overweight or obese [1]. Several studies have shown a strong association between body mass index and the risk of developing breast cancers in post-menopausal women [3-10]. On the other hand, the association between breast cancer in premenopausal women and body mass index favors a more inverse relationship [11-17] where the protective role of estrogen is thought to protect against the development of breast cancer [18-21]. Here we explore the relationship between body mass index, triple negative breast cancer and ethnicity at our inner city university hospital.

## Materials and Methods

We present a retrospective review of the Tumor Registry Database at the University of Florida, College of Medicine in Jacksonville. It involved patients diagnosed with breast cancer between 2004 and 2008. After the IRB approval, 84 patients with triple negative breast cancer were identified. Triple negative breast cancer is defined as the type of breast cancer with negative receptors for estrogen(ER), progesterone (PR), and unremarkable for the expression of HER2/Neu. For comparison, 83 control patients were randomly selected with breast cancer having at least one hormone receptors positive in the tumor profile. The data was electronically collected and later arranged into variables such as breast cancer receptor status, body mass index and ethnicity. Chi-square testing was then used to evaluate categorical variables while T-Test analysis was used for continuous variables.

### Data analysis

Our data demonstrated that 27.4% of the triple negative breast cancer patients had a BMI < 25 compared to the 14.5% of non-triple negative breast cancer, 73.6% of the triple negative breast cancer group had a BMI ≥ 25 compared to 86.5% in the non-triple negative group (p-value 0.245). This is shown in Table 1. In terms of ethnicity, triple negative breast cancer was found in 56% of African-American and 44% of Caucasians. Non-triple negative breast cancer was found in 48.2% of African-Americans and 51.8% of Caucasians (P-value 0.354) (Table 2).

**Table 1:** Comparison of BMI With Estrogen, Progesterone Receptors (ER, PR) and Her2/Neu Protein Expression

Body Mass Index (BMI)		Triple Negative Breast Cancer ( TNBC )	
		Yes (%)	No (%)
BMI < 25	Yes	27.4	14.5
	No	73.6	86.5

**Table 2:** Comparison of Ethnicity With Estrogen, Progesterone Receptors (ER, PR) and Her2/Neu Protein Expression

Ethnicity	Triple Negative Breast Cancer (TNBC)	
	Yes (%)	No (%)
African-American	56	48.2
Caucasian	44	51.8

## Discussion

Several studies have explored the relationship between body mass index (BMI) and the incidence of breast cancer [22-24]. Weight gain early on in life has also been associated with a higher incidence of breast cancer later in life [23, 25-35]. Before menopause, female ovaries play a vital role in the production of estrogen whereas in the postmenopausal stage this role is primarily taken over by the adrenal glands [36]. In premenopausal women the higher levels of estrogen results in an increased number of anovulatory menstrual cycles and therefore the higher levels of estrogen here does not lead to a significantly increased incidence of breast cancer [36, 37]. However, in the post-menopausal women with higher body mass index (BMI), higher adiposity generates elevated levels of circulating estrogen which is associated with an increased incidence of breast cancer in these females [36, 37]. Studies have also shown a higher correlation between obesity and estrogen receptor positive (ER+) breast cancers [38]. This correlation is also seen when hormone replacement therapy is used by post-menopausal women [39]. Data collected from several other studies has shown a strong correlation between obesity, higher tumor burden and higher breast cancer grade [10, 40-43] and irrespective of the menopausal status, research has also revealed poorer prognosis with higher mortality [4, 10, 41-48].

In those pre-menopausal women who do develop breast cancer, a higher BMI before diagnosis is associated with a higher mortality rate [3-10], however the association between mortality and weight increase after the diagnosis of breast cancer is not well established [11-17]. Improvement in modern diagnostic tools and therapeutic regimens have resulted in the early detection and improved subsequent management of breast cancers and this general trend is now regarded as the most significant factor in decreasing breast cancer associated mortality [49]. It is because of this reason that millions of female breast cancer survivors are alive today [49]. Besides the improved screening tools and chemotherapy regimens, special focus is also placed on cardiovascular complications which have historically been a major source of death in breast cancer survivors [50].

Body mass index is a simple tool that is often used to stratify weight status and the risk for comorbidities like hyperlipidemia, hypertension, type 2 diabetes, sleep apnea, and certain malignancies including breast cancer [51-53]. BMI is a modifiable risk factor and understanding the relationship between BMI and the aforementioned comorbidities is extremely important. Table 3 below stratifies the BMI index and the associated risk of develop comorbidities [53].

**Table 3:** Classification of Weight Status and Risk of Disease

	BMI (kg/m^2^)	Obesity Class	Risk of Disease
Underweight	< 18.5		
Healthy weight	18.5 - 24.9		
Overweight	25.0 - 29.9		Increased
Obesity	30.0 - 34.9	I	High
Obesity	35.0 - 39.9	II	Very high
Extreme Obesity	≥ 40	III	Extremely high

Our retrospect cohort study showed that the proportion of women with higher BMI and triple negative breast cancer was 73.6%. Similarly the proportion of women with higher BMI in the non-triple negative breast cancer population was 86.55%. However, the P-value was measured at 0.245, making the association between body mass index and tumor negative breast cancer to be statistically insignificant. Our findings also showed that in terms of ethnicity, triple negative breast cancer was proportionally more common in African American females (56%) compared to the Caucasian females (44%), as shown in Table 2. However, the P-value was 0.354 making this finding statistically insignificant as well.

Our findings are not in agreement with the data pooled from previous studies which not only established a higher incidence of triple negative breast cancer in African American women compared to their white counterparts, but also showed that the African American females are more likely to present at an advanced stage, higher grade and fared worse with respect to disease free survival and overall survival [54-67]. In African American women the rate of mortality from breast cancer has not only remained higher but the rate of decline has also lagged behind white women [54-65].

There are certain limitations to our research study, least of which is the small sample size. We were only able to assess 84 triple negative breast cancer patients who met our criteria in the IRB approved time period from 2004 to 2008. The small sample size makes it very difficult to deduce any significant conclusions with absolute certainty. This shortcoming can be overcome in the future by pooling data in a larger, multicenter study. Another shortcoming is that we were not able to record menstrual status in our sample size. This could have helped us further investigate the relationship between the incidence of breast cancer and menstrual status in our patient population.

### Conclusion

Our retrospect cohort study aimed to investigate the relationship between BMI and breast cancers receptor subtype and ethnicity. We were not able to show any statistically significant association between high BMI with receptor subtype or ethnicity. Our findings are not in agreement with the previously published research; however, this is a retrospective cohort study from one center with a small sample size which greatly limits our ability to deduce any definitive conclusions. In light of these shortcomings, we recommend a future multicenter study with a larger sample size.
